# Digital first primary care in NHS England: evaluating alignment with patient-centered care and implications for future practice

**DOI:** 10.3389/fdgth.2026.1723805

**Published:** 2026-05-25

**Authors:** Kunyue Xing, Jialian Wen, Dongming Xing, Bing Liang

**Affiliations:** 1 School of Public Health, Peking University, Beijing, China; 2 Alliance Manchester Business School, University of Manchester, Manchester, United Kingdom; 3 Department of Science and Technology Studies, University College London, London, United Kingdom; 4 Cancer Institute, The Affiliated Hospital of Qingdao University, Qingdao University, Qingdao, Shandong, China; 5 School of Life Sciences, Tsinghua University, Beijing, China

**Keywords:** digital first primary care, digital health equity, healthcare accessibility, hybrid care model, patient-centered care, telemedicine

## Abstract

The Digital First Primary Care (DFPC) model, introduced by NHS England, aims to enhance healthcare accessibility and efficiency by leveraging digital tools such as telemedicine, digital triage, and virtual consultations. In this structured narrative review, we synthesized UK-focused empirical, policy, and implementation literature to examine DFPC through the patient-centered care (PCC) domains of access, autonomy, shared decision-making, continuity, relational quality, and equity. The available evidence suggests that DFPC can improve convenience, flexibility, and timeliness of first contact for some patients, but these gains are unevenly distributed and depend heavily on system design, workflow integration, and patient capability. Evidence generated during the COVID-19 emergency should not be conflated with the evaluation of routine, policy-driven post-pandemic DFPC, because the goals, constraints, and patient expectations differ across these contexts. We therefore argue that DFPC aligns with PCC only when implemented within a flexible hybrid model that preserves modality choice, supports continuity, provides safe escalation to in-person care, and actively mitigates digital exclusion. Future research should prioritize patient-reported experience, continuity, safety, and equity outcomes under routine post-pandemic conditions.

## 1 Introduction

The global healthcare landscape is undergoing a transformative shift as digital technologies become integral to care delivery and patient engagement. Digital transformation in healthcare is frequently framed around telemedicine, data-enabled communication, and digitally supported service redesign intended to improve access, efficiency, and responsiveness (1, 2). In the UK, NHS England has advanced this agenda through Digital First Primary Care (DFPC), which aims to expand access through online consultation tools, remote triage, video consulting, and other digitally mediated routes into general practice (2–5). However, the patient-centered implications of this shift remain more contested than its policy ambition.

The initial expansion of remote and digital primary care was strongly accelerated by the COVID-19 pandemic, when infection control and service continuity necessitated rapid changes in consultation mode (3, 4, 6–8). Yet pandemic-era emergency remote care and post-pandemic policy-driven DFPC are not analytically equivalent. The former was implemented under exceptional conditions; the latter is a longer-term service redesign agenda linked to NHS digital access policy. This distinction matters because conclusions about access, autonomy, safety, and patient experience cannot simply be transferred from one context to the other without qualification (4, 6–8).

Patient-centered care (PCC) is commonly understood as care that is respectful of, and responsive to, individual preferences, needs, and values, and that supports meaningful involvement in care decisions (9). In digital primary care, this evaluative lens must extend beyond convenience to include modality choice, relational quality, narrative space, continuity, safety, and equity.

This article is presented as a structured narrative review. We searched PubMed, Web of Science, and Google Scholar for literature published between 2018 and 2025 using combinations of terms including “digital first primary care,” “remote consulting,” “online consultation,” “patient-centered care,” “continuity,” “health equity,” “hybrid care,” and “implementation.” We also reviewed the reference lists of key UK empirical studies and policy reports to identify additional relevant sources. We prioritized UK empirical studies, NHS and NIHR reports, patient-safety investigations, and implementation-relevant literature, while using selected international evidence comparatively where it illuminated questions directly relevant to NHS England.

**Figure 1:**
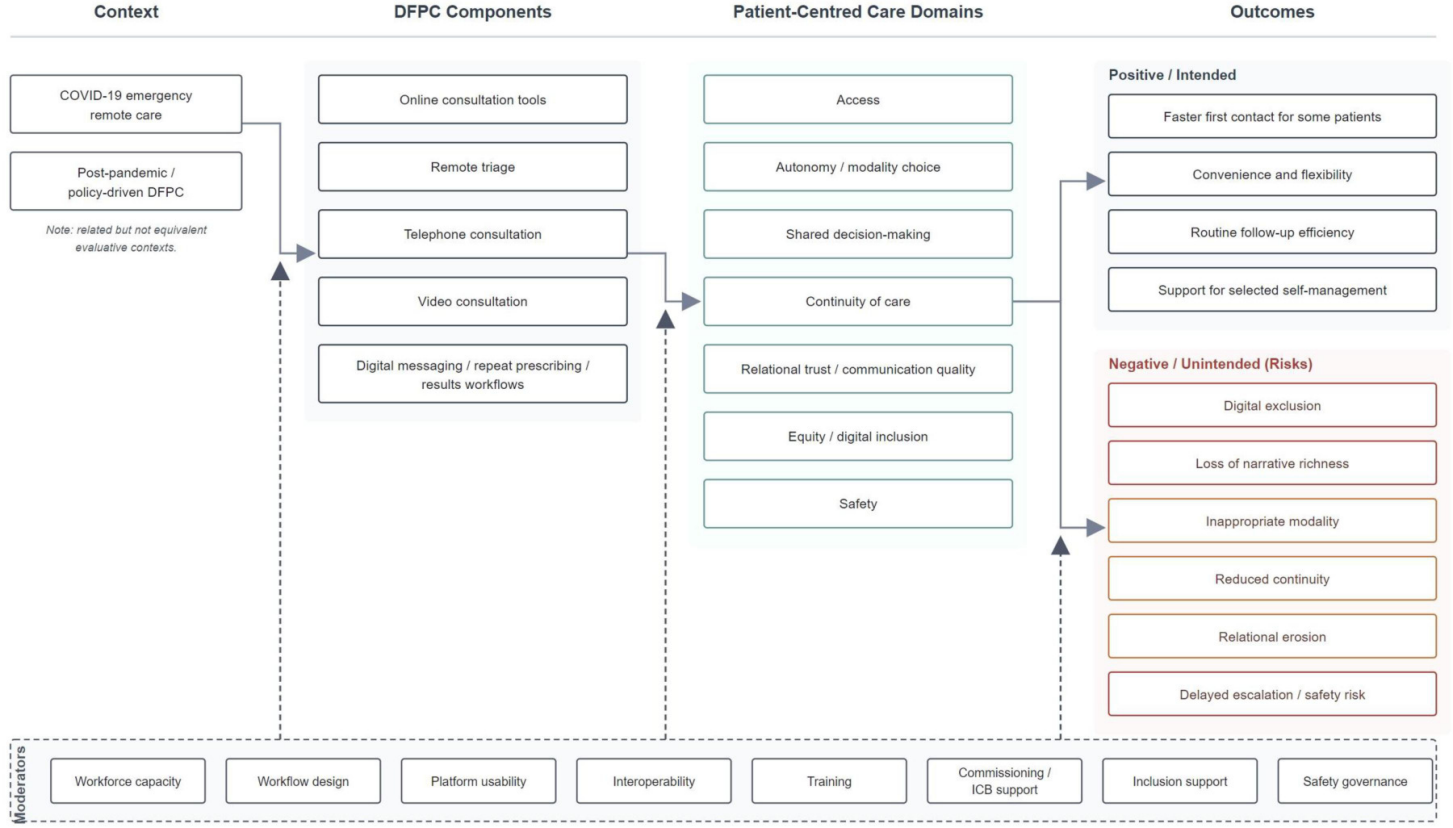
Analytical framework for evaluating digital first primary care through policy context, DFPC components, patient-centered care domains, and implementation moderators.

This review did not follow a PRISMA-based systematic review workflow and did not undertake formal risk-of-bias appraisal; instead, its purpose is interpretive, namely to assess how DFPC aligns with PCC and to identify the service conditions under which a more patient-centered model of digital primary care may be implemented. Figure 1 summarizes this analytical framework.

## 2 Digital first primary care in NHS England

### 2.1 Core features of DFPC

The DFPC model introduced by NHS England represents a strategic response to increasing demand in general practice. It uses digital tools such as online consultation systems, remote triage, telephone or video appointments, and patient-facing digital transactions to manage access and workflow (2, 4, 6, 7). However, DFPC is not a single standardized intervention. Practices vary substantially in the digital tools they use, the way online requests are integrated into daily workflow, how rapidly patients can be escalated to synchronous or face-to-face review, and the degree to which digital systems are supported by administrative and clinical capacity (6, 7, 10, 11). Core features of DFPC therefore include not only digital entry routes and remote consultation modalities, but also the organizational rules through which requests are triaged, escalated, and resolved within routine practice workflows. This means that DFPC should be understood less as a single technological intervention than as a service model whose patient-centered effects depend on local implementation.

### 2.2 Telemedicine and digital consultations

Telemedicine has transformed patient engagement by allowing consultations to take place without travel and by enabling contact to fit more flexibly around work, caring responsibilities, and other daily commitments (3, 12, 13). For selected routine, follow-up, or administrative problems, remote contact may improve convenience and reduce access friction. These advantages appear particularly relevant when digital services are used as one option among several rather than as a rigid replacement for in-person care (6, 10, 12).

However, telemedicine is not uniformly beneficial. Its acceptability depends on the clinical problem, the patient's confidence with technology, the quality of the platform, and the availability of alternative modalities. Qualitative, survey-linked, and implementation studies consistently show that digital consultations may work less well for complex, emotionally sensitive, poorly bounded, or examination-dependent concerns, and that patient experience varies by online consultation system design and by practice sociodemographic context (14–20). These limitations are especially important because they shape how telemedicine is subsequently evaluated through patient-centered care domains such as autonomy, communication, and equity (15, 16, 18).

### 2.3 Remote triage systems

Remote triage is another central component of DFPC. Patients submit symptoms or requests through online forms, messaging tools, or telephone contact, after which practice staff determine the most appropriate pathway for response (6, 7). Used well, remote triage can help practices prioritize urgent issues, route straightforward problems efficiently, and reserve in-person appointments for patients who most need examination or more complex review.

At the same time, triage is a key site of potential misalignment with PCC. Patients may struggle to summarize evolving symptoms within structured forms, and clinicians may need to make decisions without physical examination or the richer contextual cues available in face-to-face care. When the system effectively determines consultation mode without sufficiently accommodating patient preference or diagnostic uncertainty, tensions arise between operational efficiency and genuine patient choice (9, 17, 19, 21). This is particularly important in primary care, where patients often present with uncertain, evolving, or multi-problem concerns that do not map neatly onto structured triage categories.

## 3 Assessing DFPC through a patient-centered care Lens

PCC provides a useful framework for evaluating DFPC because it directs attention to more than speed or convenience alone. A patient-centered digital service should not only make contact easier, but also support understanding, involvement, trust, continuity, and equitable access across different patient groups (9, 22).

### 3.1 Autonomy and empowerment in digital health

Digital platforms may empower patients by enabling them to request prescriptions, book appointments, access information, view records, and manage elements of care at times that suit them (10, 12, 22). For many patients, this flexibility represents a meaningful enhancement of autonomy and responsiveness.

Yet autonomy should not be treated as an automatic consequence of digitization. A core paradox of DFPC is that a pathway designed to increase convenience can reduce autonomy when patients are channeled toward digital routes they did not choose, do not trust, or cannot use effectively. Genuine autonomy therefore depends not simply on digital availability, but on modality choice, accessible design, assisted digital support, and clear escalation routes to telephone, video, or in-person care when needed (10–12, 15, 17). In this sense, autonomy in DFPC should be judged not by digital availability alone, but by whether patients retain meaningful agency over how they enter, navigate, and escalate care.

### 3.2 Challenges in PCC integration with DFPC

Two particularly important challenges to PCC integration in DFPC concern digital exclusion and communication-related constraints on shared decision-making. One of the most pressing PCC challenges is digital exclusion. Older adults, people with limited digital literacy, those with language or cognitive barriers, and patients facing material deprivation may all encounter disproportionate difficulty in navigating digitally mediated access (15, 16, 18, 23). Importantly, exclusion is not limited to device ownership; it also includes confidence, usability, trust, comprehension, and the availability of acceptable alternatives (10, 11, 15, 16, 18).

A second challenge concerns communication and shared decision-making. Some patients feel that online systems constrain how they can tell their story, while clinicians may find it harder to assess complexity, uncertainty, risk, or emotional context without richer interaction. In such circumstances, DFPC may undermine shared decision-making by narrowing the patient's opportunity to explain concerns fully or to influence how care is delivered (9, 19–21).

### 3.3 Patient voice and lived experience

Patient voice is central to judging whether DFPC is truly patient-centered. Recent English qualitative studies show that many patients value the speed, flexibility, and practicality of online or remote access for straightforward concerns. At the same time, they report difficulty expressing complex problems in structured digital forms, uncertainty about whether their concern has been fully understood, and frustration when digital routes feel mandatory rather than optional (12, 17). Survey-linked English evidence further suggests that the relationship between online consultation use and patient experience varies by system design and by practice sociodemographic characteristics, reinforcing that acceptability is shaped by both technology and context (14). These accounts also indicate that patients evaluate digital access not only in terms of speed, but also in terms of whether they feel heard, understood, and appropriately supported within the interaction. For some patients, repeated navigation of digital routes may itself become effortful, especially when systems are perceived as administratively efficient but relationally thin.

These studies also suggest that acceptability is contingent rather than universal. Remote and online care may work well when the issue is familiar, bounded, and low risk, or when the modality reduces stigma, travel, or time burden. By contrast, patients often prefer face-to-face care for symptoms that are hard to describe, emotionally sensitive, potentially serious, or perceived to require examination. This qualitative literature strengthens the view that PCC alignment cannot be inferred from operational efficiency alone (12, 17, 19, 20).

### 3.4 Patient-Perceived benefits under appropriate conditions

These concerns should not obscure the fact that DFPC can still generate meaningful patient benefits under appropriate conditions. NHS and NIHR evidence suggests that digital-first models may enable some patients to reach a clinician more quickly than traditional routes, particularly for routine, administrative, or follow-up issues and in some contexts involving multiple long-term conditions (6, 7, 11). However, such gains are conditional and should not be generalized as fixed effect sizes across all patient groups or service settings.

Remote care may also reduce practical burdens associated with travel, waiting rooms, time away from work, or repeated low-value visits. For some patients, especially when relational continuity is preserved and escalation remains straightforward, this can improve the overall experience of care. The patient-centered value of DFPC therefore lies not in replacing in-person care, but in using digital access selectively and responsively (3, 12, 13).

## 4 Implementation implications and the case for hybrid care

### 4.1 Why hybrid care is more compatible With PCC

Taken together, the evidence suggests that DFPC is most compatible with PCC when implemented as part of a flexible hybrid model rather than as a predominantly digital-only gateway. A hybrid approach preserves digital, telephone, video, and in-person routes and allows consultation mode to be matched to patient preference, clinical appropriateness, and diagnostic uncertainty. The contrast between a predominantly digital-first model and a PCC-oriented hybrid model is summarized in Table 1 (6, 20, 24).

**Table 1:** Comparison of DFPC and a PCC-oriented hybrid model.

Domain	Predominantly digital-first model	PCC-oriented hybrid model	Practical requirements for implementation in NHS England
Entry route to care	Digital route may become the default gateway; efficient for some but restrictive for others	Multiple legitimate access routes retained, including online, telephone, and in-person options	Reception and triage protocols, protected non-digital routes, clear patient-facing communication
Patient autonomy and choice	Convenience may increase for confident users, but actual modality choice may narrow	Consultation mode selected through patient preference plus clinical appropriateness	Explicit modality-choice policy, transparent escalation rules, clinician discretion
Shared decision-making	Structured forms may compress the patient narrative and reduce deliberation	Digital tools used to support, not replace, collaborative discussion	Better form design, free-text capacity, decision aids, easy conversion to live contact
Continuity of care	Continuity can be weakened when triage disperses care across multiple clinicians	Remote and in-person follow-up linked to a known clinician or team where possible	Named-clinician workflows, team continuity rules, follow-up allocation protocols
Equity and inclusion	Higher risk of exclusion for digitally disadvantaged patients	Digital and non-digital routes combined with targeted inclusion support	Assisted digital support, language access, accessible interface design, subgroup monitoring
Clinical assessment and safety	Risk of incomplete assessment, inappropriate modality, and delayed escalation	Modality matched to uncertainty, complexity, and need for examination	Safety-netting standards, escalation thresholds, incident review, clinician training
Workforce impact	May shift rather than reduce workload; inbox burden and duplicated contacts possible	Workflow deliberately redesigned across administrative, nursing, and GP roles	Capacity planning, protected triage time, adequate administrative staffing, role clarity
Commissioning and infrastructure	Procurement may prioritize access metrics over usability or safety	Procurement aligned with interoperability, usability, safety, and inclusion	ICB/practice co-design, interoperable systems, stable funding, evaluation requirements
Training and normalization	Staff may be expected to adapt without consistent preparation	Hybrid care embedded through training, feedback, and iterative refinement	Remote-consulting training, communication skills development, implementation leadership, audit and learning

### 4.2 Implementation requirements for hybrid care

This recommendation, however, is not merely aspirational. Hybrid care requires deliberate implementation work: workforce capacity to process online demand safely; commissioning and infrastructure that support usable and interoperable systems; training in remote communication and triage; practical digital facilitation for patients who need help navigating access; and local safety oversight to monitor implementation risks and unintended consequences (10, 11, 15, 23–27). If these conditions are absent, digital expansion may simply redistribute burden to patients, reception staff, and clinicians rather than improving care.

Implementation science offers useful frameworks for this task. Consolidated Framework for Implementation Research (CFIR) helps identify determinants across the intervention, inner setting, outer setting, individual actors, and implementation process, while Normalization Process Theory (NPT) helps explain whether hybrid workflows can become embedded in routine practice (23, 26, 27). Read through these lenses, hybrid care is not simply a preferred endpoint; it is a sociotechnical service model that must be actively designed, resourced, monitored, and refined.

## 5 Review limitations

This review has several limitations. First, it is a structured narrative review rather than a systematic review; it does not provide a PRISMA flow diagram, exhaustive record counts, or formal risk-of-bias assessment. Second, the evidence base on DFPC remains heterogeneous, spanning empirical studies, service evaluations, policy documents, patient-safety investigations, and implementation reports with differing methodological strengths. Third, although this review is centered on NHS England, some international literature was used comparatively where UK evidence was limited. Finally, a substantial proportion of published evidence on remote and digital primary care derives from the COVID-19 period, which complicates direct inference about routine post-pandemic DFPC (8). These limitations reinforce the need for further patient-focused and implementation-focused research, particularly under routine post-pandemic conditions in NHS England.

## 6 Conclusion

This review suggests that the central question is not whether DFPC expands access, but under what conditions it does so in a genuinely patient-centered way. DFPC appears most defensible when it operates within a carefully designed hybrid model that preserves modality choice, supports continuity, enables safe escalation, and actively reduces digital exclusion. Future evaluations should move beyond headline efficiency claims and examine patient-reported experience, continuity, safety, and equity under routine post-pandemic conditions.
